# Healthcare workers’ perceptions and experiences of implementing voluntary medical male circumcision in KwaZulu-Natal, South Africa

**DOI:** 10.4102/phcfm.v12i1.2253

**Published:** 2020-03-25

**Authors:** Celenkosini T. Nxumalo, Gugu G. Mchunu

**Affiliations:** 1 KwaZulu-Natal Department of Health, Ndwedwe Community Health Centre, Ndwedwe, South Africa; 2 Discipline of Nursing, School of Nursing and Public Health, University of KwaZulu-Natal, Durban, South Africa

**Keywords:** voluntary medical male circumcision, HIV prevention, health care workers’, perceptions, experiences, service implementation

## Abstract

**Background:**

Voluntary Medical Male Circumcision (VMMC) is an effective HIV prevention strategy prioritized by the World Health Organisation (WHO) for regions of high HIV prevalence, South Africa (SA) and in particular KwaZulu-Natal (KZN) is one of such regions. Since the roll out of VMMC in 2010 there has been little research conducted on the implementation of this service. Existing studies on the uptake of VMMC have mainly focused on service users resulting in a paucity of data on health care workers perspectives on the intervention.

**Aim:**

To analyse health care workers’ perceptions and experiences of implementing voluntary medical male circumcision in KZN, SA.

**Setting:**

The study took place at six different health districts and their six respective rural clinics in the KZN province of SA.

**Methods:**

A qualitative approach using a phenomenographic design was employed. Data were collected from a sample of 18 participants comprising of health care providers (*n* = 12) and health policy makers (*n* = 6). Individual, face-to-face interviews were conducted using a semi-structured interview guide. An audiotape was used to record the data, which were transcribed verbatim and then analysed using a step-wise phenomenographic data analysis procedure.

**Results:**

Participants reported that VMMC was implemented by the department of health with support from non-governmental organisations and private general practitioners. Negative perceptions and negative experiences regarding VMMC and implementation were reported.

**Conclusion:**

The implementation of VMMC is compromised due to poor preparation and training of healthcare workers for implementing the service. Addressing health care workers’ needs for training and preparation is crucial for successful implementation of VMMC.

## Introduction

Medical male circumcision (MMC) reduces the chances of heterosexual transmission of HIV infection by up to 60%.^1,2,3^ Countries with high HIV prevalence have adopted voluntary MMC as a preventive measure against HIV as recommended by the World Health Organization (WHO). One such country is South Africa (SA), the KwaZulu-Natal (KZN) region in particular.^4^

Male circumcision was reintroduced medically in SA in 2010 after being abolished by the king of the Zulu tribe in 1780, because of complications that prevented men from engaging in war.^5,6^ Since the roll out of voluntary medical male circumcision (VMMC), there have been concerted efforts to implement MMC for 80% of males between the ages of 15 and 49 in order to prevent new HIV infections.^7,8,9^ By the end of 2018, approximately 1.2 million medical circumcisions had been conducted in KZN. In spite of this significant effort, the number of males circumcised within the targeted age group remains well below the target of 80%.^9,10^

Research on the scale-up of VMMC in older men reveals that several factors need to be taken into consideration when attempting to increase the number of males undergoing the procedure in the target group. The factors include individual perceptions, and the influence of culture, religion, societal norms, close peers and female partners.^11,12,13^

The health system’s ability to render VMMC to meet the demands, in terms of both the quality of care and the needs of men, is also a factor to be considered.^14^ In this regard, appropriate policy and implementation strategies at all levels of care in the healthcare system are necessary if an effective VMMC service is to be delivered.^15^

The implementation of VMMC in KZN, SA, and other areas of sub-Saharan Africa has largely been guided by local policies and strategies using operational guidelines developed by the WHO and The Joint United Nations Programme on HIV and AIDS (UNAIDS). The successful implementation of VMMC to help achieve stipulated targets is dependent upon the understanding of the contextual factors that influence or hinder the uptake of VMMC. Although research has been conducted on the uptake of VMMC in KZN, SA, it has been oriented mainly towards service users in terms of understanding the individual range of barriers and facilitators for service uptake.^16,17^ In addition, most of such research has been conducted on adolescents.^16,18^

To date, there has been little empirical research conducted on existing practices of implementing VMMC services at the primary care level; moreover, the contextual barriers and drivers for VMMC in the adult male population have not been studied adequately from the perspective of healthcare workers.^19^

The present study was conducted to analyse healthcare workers’ perceptions and experiences of implementing VMMC in KZN, SA, in order to elucidate the current implementation practices, challenges, experiences, and perceived barriers and drivers for uptake. The objectives of the study are to determine the current practice of implementing VMMC in KZN, SA, and to analyse healthcare workers’ perceptions and experiences of implementing VMMC in KZN, SA.

## Methods

### Study design

A qualitative approach using a phenomenographic design was used to analyse healthcare workers’ perceptions and experiences of implementing VMMC in KZN, SA. The participants of the research study were healthcare providers involved with the implementation of VMMC and policymakers at the different district offices.

Phenomenography is a research approach that seeks to identify and describe ways in which a group of people experience, interpret or perceive an aspect of reality.^20^ As a design, it arises from a study conducted by Ference Marton meant to understand how students experienced and understood learning.^21^ In recent years, the design has been used in health sciences, particularly in medical and nursing education, to generate knowledge of service users’ experiences of care.^20,22,23^

This study was part of a larger study to analyse primary healthcare (PHC) stakeholders’ experiences, understanding and conceptions of VMMC in KZN, SA, in order to develop a relevant intervention to support the existing VMMC implementation strategy. Data were collected from 07 January to 30 March 2019.

### Setting

This research was conducted at six different health district offices and respective PHC facilities in KZN, SA. The health districts that were included were Ilembe, Umkhanyakude, Harry Gwala, Zululand, Umzinyathi and Uthukela. All the selected districts offered VMMC services and have an estimated population of between 110 000 and 250 000 men in the age group of 15–49 years. According to the KZN Department of Health District Health Information System (2018), approximately 5000–19 000 VMMC procedures have been performed in these districts in the recent years. Each district has about 35–72 PHC facilities, which have recently performed around 700–2800 VMMC procedures. Just over 60% of these VMMC’s have been conducted on boys aged 10–14 years, indicating that these districts continue to fall short of VMMC in the target age group of 15–49.

### Sampling and recruitment strategies

Health districts and PHC facilities were sampled purposively, based on the sociocultural diversity and performance of VMMC per district and facility. Demographic information regarding the target population serviced by all health districts was obtained from all the health district offices in KZN. The selection of the districts for data collection was then made on the basis of the number of VMMC procedures performed annually and those districts that were most representative of the general KZN population of males in terms of ethnicity, and cultural, religious and traditional diversity. Purposive sampling of healthcare service providers and policymakers was also conducted; only healthcare workers who had been involved in the implementation of VMMC were recruited to be part of the study.

The data for healthcare service providers were collected from six different clinics that had been identified from the selected health districts; for policymakers, the data were collected at the respective health district offices. Both groups of participants were recruited through mediated access, which entailed an initial briefing of the participants’ immediate supervisors regarding the nature of the study. Initial data analysis was conducted concurrently with data collection; recruitment and interviewing ceased once data had been saturated based on the analysis that was conducted as the researcher went along with data collection. Of the 18 participants included in this study, 12 were health service providers and six were policymakers. The healthcare providers were interviewed individually in private consulting rooms after they had completed their duties, and the policymakers were also interviewed privately when it was convenient for them.

For the purpose of this study, healthcare workers refer to healthcare service providers and policymakers who comprise the two population groups of this study. Healthcare providers refer to individuals involved in the provision of health services at the PHC level, namely, all categories of employed nurses, medical doctors and clinical associates. For this study, policymakers included the individuals working in the Department of Health who were responsible for the formulation of policies, strategies and protocols based on which VMMC and other health-related services must be rendered.

### Data collection

The data were collected through individual in-depth interviews guided by a self-developed interview schedule. A pre-test was conducted with one healthcare worker to validate the data collection instrument before the actual study was conducted. There were no changes made to the original instrument, as it was found to allow for an in-depth enquiry into perceptions and experiences related to the implementation of VMMC.

The data collection instrument consisted of two sections – the first was related to socio-demographic data and the second to guiding interview questions relating to participants’ perceptions and experiences of implementing VMMC in KZN, SA. Interviews were conducted in English for all participants. Interviews for healthcare workers were conducted at the selected clinics of the six health districts whilst interviews for policymakers were conducted in the district offices. An audiotape was used to record all interviews, which ranged from 25 to 45 min in duration.

### Data analysis

The data collection interviews were transcribed verbatim and were then analysed in a step-wise manner following phenomenographic data analysis procedures. The data analysis was iterative, using a step-wise approach. The first step was to read the interviews repeatedly to gain an overall insight into the data. Statements relevant to the research questions were identified in the transcripts. These statements were re-read, summarised and grouped according to categories of similarity and difference. These categories were then compared to establish the borders between them. Finally, categories were named and refined. A constant process of iteration with emphasis on whole and part statements in the data was used, until identified descriptive categories were found to be mutually exclusive.

### Trustworthiness

To ensure trustworthiness, data analysis was conducted in collaboration with an expert (the research supervisor) while employing qualitative research methods. To ensure richness of the data, individual in-depth interviews were conducted; preliminary data analysis was conducted along with data collection, and interviews ceased when data had been saturated. To ensure reliability and credibility of the findings, multiple reviews of voice recording were conducted in comparison with the transcribed data.

### Ethical consideration

This study forms part of a larger study conducted to analyse the qualitative differences in primary healthcare stakeholders’ experiences, understanding and conceptions of voluntary medical male circumcision in KwaZulu-Natal. Ethical approval to conduct this study was obtained from the Biomedical Research Ethics Committee of the University of KwaZulu-Natal (BE 627/18). Informed consent was obtained verbally and in writing prior to data collection from the participants. A written information sheet was provided to the participants to clarify the nature of the study, and written consent was sought after obtaining verbal approval by the participants. Ethical clearance was obtained from the Biomedical Research Ethics Committee of the University of KwaZulu-Natal on 10 December 2018, and recertification was granted on December 2019 with the ethical clearance number: BE627/18.

## Results

### Socio-demographic data

A total of 18 participants formed part of the study. The study participants included healthcare service providers (*n* = 12) and health policymakers (*n* = 6). Healthcare providers had been involved in the implementation of VMMC at the facility level for a minimum of 6 months. The healthcare providers were located predominately in rural areas of KZN, SA. Health policymakers were from four different health districts and had been involved in VMMC policy development and implementation for a minimum of 2 years. The demographic details of participants are detailed in Table 1.

**TABLE 1:** Profile of participants.

Participant	Unique Code	Designation	Years of experience with VMMC implementation
Policy maker1	PM01	District VMMC clinical programme coordinator	6 months
Policy maker2	PM02	District HIV/AIDS programme coordinator	4 years
Policy maker3	PM03	District VMMC clinical programme coordinator	18 months
Policy maker4	PM04	District family physician	6 years
Policy maker5	PM05	District HIV programme coordinator	3 years
Policy maker6	PM06	District VMMC clinical programme coordinator	2 years
Healthcare worker1	HCW01	Professional nurse	2 years
Healthcare worker2	HCW02	Professional nurse	1 years
Healthcare worker3	HCW03	Medical doctor	3 years
Healthcare worker4	HCW04	Clinical associate	8 months
Healthcare worker5	HCW05	Clinical associate	1 year
Healthcare worker6	HCW06	Professional nurse	18 months
Healthcare worker7	HCW07	Enrolled nurse	9 months
Healthcare worker8	HCW08	Enrolled nurse	1 year
Healthcare worker9	HCW09	Medical doctor	4 years
Healthcare worker10	HCW10	VMMC community mobiliser	12 months
Healthcare worker11	HCW11	Nursing assistant	8 months
Healthcare worker12	HCW12	Professional nurse	3 years

VMMC, Voluntary medical male circumcision; HIV/AIDS, human immunodeficiency viruses/ acquired immunodeficiency syndrome.

The findings of this study reveal several categories of description in participants’ perceptions and experiences of implementing VMMC in KZN, SA. The categories of description are outlined in Table 2.

**TABLE 2:** Categories of description in experiences and perception regarding implementation of VMMC in KZN.

Category 1 (Emerging categories of perceptions)	Category 2 (Emerging categories of experiences)
**Practice of implementing VMMC** **Perceptions about the impact of MMC** Improved health outcomes Possible risky behaviour Mixed feelings **Perceived barriers uptake of VMMC** Poor mobilisation of VMMC Negative health user perceptions Poor coordination of services **Perceived drivers of MMC** Persistent demand creation for VMMC Integration of cultural practices into VMMC	**Role of the Department of Health staff in VMMC implementation** Poor preparation of healthcare workers Service delivery issues Lack of ownership by the Department of Health staff

VMMC, Voluntary medical male circumcision; MMC, medical male circumcision; KZN, KwaZulu-Natal.

#### Practice of implementing voluntary medical male circumcision

From the policy makers’ perspectives, the facility for VMMC is provided by non-governmental organisations (NGOs) at the PHC level, with the Department of Health staff providing a coordinating service encompassing provision of facilities, counselling and follow-up post-VMMC surgery. At the facility level, VMMC was conducted through mass campaigns and same-day services. The national guidelines on VMMC and other PHC guidelines form the guiding framework upon which services are rendered at an operational level. Certain healthcare workers reported that, in some facilities, VMMC was rendered exclusively by the NGO partners, rather than by the staff working for the Department of Health:

‘I think it’s done almost exclusively by the Non-Governmental Organisation (NGO) partners. You know most of it now I think is done by the partners rather than by the healthcare providers.’ (PM04)

‘We have Medical Male Circumcision (MMC) camps which are done in collaboration with the supporting and NGO like once a month or even twice if there is [*a*] demand and then it’s the daily MMCs which are done by the clinical associate or doctor or trained nurse.’ (PM01)

‘We have partner NGOs and contracted General Practitioners who are meant to work with staff within the department of health to render daily MMCs on demand and camps which are usually done on school boys.’ (PM02)

#### Role in voluntary medical male circumcision implementation

Healthcare workers reported that they mainly play an administrative support role in terms of implementation, coordination follow-up and counselling regarding VMMC. Policymakers stated that they were involved mainly in overall strategy, coordination and planning in terms of delivery of the service:

‘I am responsible for [*the*] overall running of the programme in the entire district; I am responsible for planning and organisation of the programme and ensuring that facilities have enough equipment and resources to render services. I also intervene during adverse events and ensure that overall we comply with the prescribed guidelines to render MMC.’ (PM06)

‘In this facility, I am responsible for running the programme organising MMC camps and ensuring that the supporting partner has everything they need during a camp. I also deal with patients after they have done MMC; assess wounds and prescribe the relevant treatment if necessary and provide health education.’ (HCW01)

‘I work to mobilise boys and certain men to undergo MMC and also during the procedure, I provide counselling to patients and do preoperative preparations like injecting them before they undergo the actually foreskin removal.’ (HCW08)

### Experiences

#### Poor preparation of healthcare workers

Most participants reported that they were not fully prepared for the role that they were playing in terms of implementing VMMC. The policymakers reported that they received little formal training to equip them to carry out their responsibilities effectively. Healthcare service providers, mainly nurses, reported that they were not trained regarding VMMC both in terms of theory, clinical practice and service implementation in relation to existing policies and guidelines:

‘The challenge here is that I am working as an infection, prevention and control (IPC) coordinator and have to run MMC in the facility, and I have not received any theoretical or practical training. It is only the clinical associates that have been trained. I have to do everything based on the knowledge I have obtained from asking around and reading on my own.’ (HCW12)

‘I am coordinating MMC, HIV prevention and the community care giver (CCG) programme[*s*] and while I have worked in the area[s] of MMC and HIV before, it’s different from what I have to do here because it involves coordination of so many programmes at such a high level. There was no preparation I received for how one actually goes about coordinating programme[*s*] like this one from the position that I am in.’ (PM05)

‘Even though I have an idea of how MMC is done, as a female it’s difficult to run a male programme like this and within the department there is no preparation for how you deal with such and how you navigate as a female.’ (HCW02)

#### Service delivery issues

The participants reported challenges relating to the rendering of the VMMC service within the context of PHC. These challenges were related to the inadequacy of the working environment to meet the demands of circumcising a large number of men and boys on occasions when the demand for VMMC had increased. In addition, the lack of sufficient skilled personnel was also a reported challenge, as it significantly impeded the postoperative follow-up and management of the surgery:

‘When there are camps, there are large camps; the challenge sometimes is getting the space to perform the procedure.’ (HCW07)

‘Follow-up, post-operatively, is usually a problem particularly the second and third follow-up. When you contact the NGO who ha[s] done the circumcisions, they are sometimes not available as they have been booked at other sites.’ (HCW12)

‘I am the only one who is trained and responsible for MMC. When I am not around, there is no one to render services and to follow-up or manage boys who present with MMC-related problems.’ (HCW05)

#### Lack of commitment and ownership by the Department of Health staff

The policymakers reported that there was poor sustainability of the programme in terms of service delivery, particularly among staff who were working within the Department of Health. These participants also reported that the staff working within the department and the supporting private sector staff did not adhere to the concepts of prevention and health promotion when providing VMMC services:

‘I find that one of the problems is that staff within our department rely too much on the supporting partner and end up not being interested or involved in MMC.’ (PM01)

‘The problem is that those rendering services have lost the conception of prevention and promotion, so they just remove the foreskin and move on.’ (PM02)

‘The NGOs are not interested in comprehensive management; they are just chasing heads and looking for foreskin[*s*] because a certain number of circumcisions equals a specific amount of money. As a result, we lose the effectiveness of the intervention.’ (PM05)

#### Distorted communication and poor role clarification

The participants reported that there were poor reporting systems and channels of communication between staff working within the Department of Health and those from the private sector. The consensus among participants was that the role of each party was not clearly defined by existing policies and guidelines. The healthcare providers at the PHC level reported that they lacked guidance on all forms of reporting pertaining to VMMC services; moreover, there was no clarity as to who should report and when. In certain instances, there was under-reporting of data and in some cases duplication, because there was no structure in this regard:

‘The statistics for MMC reported by health facilities and the NGO partners often contradict one another. Both the facilities and supporting partners submit a report on performance every month, but at times you will have duplication of data. This arises during MMC camps where an NGO will come and do MMC and a private GP [*also*], thereafter both [*will*] report on the same camp[.] There is [*also*] no proper regulation as to who come[*s*] when and how. All these partners are just chasing boys so they just appear anyhow, do circumcisions and report on them and then expect to be paid, so there are huge problems.’ (PM02)

‘I don’t know what role the NGO plays in this whole MMC thing because at times they will come during the camp and say they do not have enough staff so they need our staff to help them. We then have [*to*] release staff to work with them. Almost all the time we must provide them with someone to do HIV testing and help them to perform the actual procedure. At times, they will not even have sufficient equipment which we must provide.’ (HCW12)

‘The department has a Memorandum of Understanding [MOU] with supporting NGOs but from what is currently happening on the ground with GPs, NGOs and personnel in the departments – it’s like people are not aware of their responsibilities. I think that while the boundaries have been set, they are not clear enough for all those involved with implementation of MMC at the lower level[*s*].’ (PM05)

### Perceptions of staff about the impact of medical male circumcision

#### Improved health outcomes

The participants were of the view that VMMC improved the health of men, as it reduces the chances of HIV transmission and has indirect benefits for females. Some of the participants also alluded to the improved hygiene that stemmed from medical circumcision:

‘Medical circumcision is a good procedure because it reduces the chances of HIV infection by up to 60%. That is significant protection when you look at how much one is at risk of infection because the HIV rate is so high.’ (HCW03)

‘The procedure has a lot of benefits, among them being improved hygiene, prevention of certain STIs [*sexually transmitted infections*] and HIV. Women also indirectly benefit when a man is circumcised.’ (HCW04)

#### Possible risky behaviour

Certain participants expressed concern that as some men could misunderstand the partial protection offered by VMMC against HIV infection; this could result in risky sexual behaviours. This would have negative implications on their health and could potentially increase the burden of HIV infection, thus making such interventions futile:

‘I think we need to be careful with this one because some of them may think that they are completely protected from HIV and engage in risky behaviour.’ (HCW07)

‘The concern I have is that some men may think that they are immune to HIV because we are hearing messages that medical circumcision protect[*s*] against HIV. I think these campaigns must make it a point that there is no complete protection once you circumcise.’ (HCW06)

#### Mixed feelings

A significant number of the participants reported having reservations about the nature of the procedure. While they expressed belief in the efficacy of VMMC to reduce HIV infection and other associated infections, they had doubts about how the procedure was performed, especially about its safety in relation to the potential complications of VMMC surgery. Some also felt that such a service undermined the ability of men to practice responsible sexual health behaviours:

‘Well it’s an operation that is not completely risk free. Even the doctors’ hands are not completely risk-free… you can get quite a lot of bleeding.’ (HCW11)

‘I have not done it myself and I don’t think that I need to because I do not think I am living a lifestyle that requires it.’ (PM04)

‘I have slightly mixed feelings about it … well, studies have shown that it protects you, but it’s lots of work on the part of health services and it’s saying that we just cannot rely on you men to be responsible.’ (HCW09)

### Perceived barriers to uptake

#### Poor mobilisation of voluntary medical male circumcision

Most participants believed that the main barrier to the uptake of VMMC, particularly in the projected age group, is its inability to create a demand for the service. The policymakers, in particular, reported that healthcare workers do not provide comprehensive information suited to the needs of individual men. Moreover, they believed that healthcare workers had lost the concept of health promotion, meaning that healthcare workers failed at providing consistent and relevant information pertaining to VMMC:

‘There isn’t enough recruiting done by those working at health facilities. It is important for healthcare workers to provide information consistently and actually be going out to the people at community level. Right now, I think adequate dissemination on information is lacking.’ (PM01)

‘The problem we have is that we only have one person mobilising men and boys for us; as a result, we are not reaching enough and relevant people.’ (HCW02)

‘I think certain healthcare workers lack [*a*] belief in health education and how it should be done and [*that*] is lack also [*exists*] in terms of communicating the relevant information pertaining to MMC. If this change[d], then I think performance would not be the way it is now.’ (PM05)

#### Negative health user perceptions

Most participants stated that the one of the major barriers to uptake was the misperceptions, arising from the influence of tradition, religion and society, that men hold towards VMMC. It was also reported that the nature of traditional circumcision, its history in KZN and the current religious practice of circumcision (such as in the Shembe traditional religion) also had an influence on the way in which men perceived the medical approach to circumcision. Some men have misperceptions stemming from the nature of traditional circumcision, which causes feelings of anxiety and fear related to complications, while others perceive the traditional approach to be superior because of the traditional connotations associated with it:

‘I think they are probably scared of the operation; they fear pain and other complications that may come with the procedure.’ (PM01)

‘There are these stories and rumours that go around about the procedure and treatment. Some will have a bad experience and those go around like the cases with the implanon contraceptive.’ (PM04)

‘The problem is that young men generally don’t like seeking healthcare until they are sick and that goes back generally [*to the*] way in which men have been cultured. Another factor is that this MMC thing is very much a private male thing and when men see a woman involved in it, it’s a real turn-off. I have seen this personally.’ (PM06)

#### Poor coordination of services

The participants reported that at times the model of VMMC delivery often resulted in too many people doing the same thing, thus making it impossible to track progress. Cooperation between the stakeholders was poor because their roles were not clearly defined. In addition, there would be instances where services would not be rendered to patients, especially during VMMC camps, because of a lack of agreement and understanding of the roles of the parties involved. The simultaneous involvement of many supporting private-sector partners often resulted in disagreements, poor communication, lack of collaboration and, subsequently, poor management of the patients, particularly during the postoperative period:

‘The department has many supporting partners and in certain district[*s*] there are more than one and this creates a problem when MMCs are being done because, in certain instances, a particular NGO poaches [*the*] clients of another NGO, resulting in conflicts among them and confusion with staff working for the Department of Health. In such cases, there is over-reporting of statistics and problems arise with finances because both want to get paid.’ (PM02)

‘This whole MMC has become a mess because it’s now become like a money-making scheme and the Department is to blame for this. They have contracted NGOs and private doctors who have turned this into [*a*] war for foreskins, so you will now find a doctor coming from his practice, doing MMCs in an area he is not supposed to and then wanting to claim for it. In certain instances, during camps, a GP may come and take away patients while they are waiting to be circumcised, do them in his practice when he has not mobilised for them and that creates problems’. (PM05)

‘I have faced several issues in this programme, most as a result of people coming into the district and facilities claiming that they [*are*] from the Department, mobilising people and circumcising them and then not doing follow-ups, and the[*n*] when there are complications, they are nowhere to be found.’ (PM06)

### Perceived drivers of medical male circumcision

#### Persistent demand creation for voluntary medical male circumcision

Most participants reported that consistent, targeted messaging regarding VMMC is essential to promote the acceptability and the uptake of VMMC. To create a demand for this service, the participants emphasised the importance of engaging stakeholders and using structures at the community level, which included traditional and religious leaders, as well as using available public forums and media to create awareness. The use of ward-based outreach services and school health teams was highlighted:

‘The essential thing is having adequate mobilisers who are both well-informed about medical circumcision and who people can identify [*with*] and relate [*to*].’ (PM03)

‘Healthcare workers need to be persistent in providing information about medical circumcision and must always be marketing the service to all males they come across.’ (PM05)

‘The key is addressing the negative mindsets that people have regarding MMC. It’s not just about providing information but [*one*] also needs to engage with people who are influencers at community level such as your chiefs, pastors and so forth. [*If*] we can get buy-in from them, then we can win because they are the ones who know the people and actually have more of a voice.’ (PM02)

#### Integration of cultural practices into voluntary medical male circumcision

Certain participants stated that men would accept the MMC procedure if it was rendered in a manner that was identifiable to them in terms of their norms as well as their general belief system. When alluding to this, some participants suggested gathering men in a forum and carrying out traditional customary activities for men prior to any health-related activity as a means of engaging with them to obtain buy-in. Another suggestion was considering men who they could relate to from a traditional perspective, or those who had already undergone medical circumcision. Participants also reported that allowing men to be at the forefront of everything that pertained to VMMC at every level could be instrumental in creating acceptability, particularly in areas where there were reported issues related to gender and the uptake of medical circumcision by men:

‘One thing that I have observed over the years working with MMC, and in particular with men, is that they are eager to listen to something from someone they can relate [*to*]. By that, I mean like a male figure who is from their community, who knows and understand[*s*] these issues of manhood. I have found that communities are often resistant when someone who is an outsider comes to introduce something that is foreign to them.’ (PM02)

‘When you address men, it is important that you are aware of their cultural and traditional belief system. Therefore, when approaching them with a matter that is of such cultural significance, you do not just enter and start talking your health stuff. It first starts from when you gather them. You cannot expect real men to assemble at a hall; they will know you want to preach something to them and will not be interested. Instead, you call them for a feast of some sort, allow them to engage in these manly customary activities of a feast; [*while*] they are doing that, you are engaging in conversations about manhood, etc., and then somehow you find a way to subtly bring in your health part.’ (HCW10)

## Discussion

The findings of this study indicate that VMMC is implemented by the Department of Health with support from NGOs. Participants reported negative perceptions and negative experiences regarding the implementation of VMMC, owing to poor preparation and training.

This research study found that the implementation of VMMC in KZN was conducted with support from private sectors provided by contracted NGOs and private general practitioners. This is similar to the practice of implementing VMMC elsewhere, which has been possible with support from different stakeholders.^24^ The benefit of multi-sectoral collaboration for the implementation of VMMC is that it ensures that greater coverage of VMMC is attained in the stipulated target group and that more skills and resources are available to meet healthcare demands.^25^

It was found that staff members of the Department of Health were mainly involved with coordinating MMC, conducting follow-ups and counselling at an operational level. On the other hand, the role of policymakers in the implementation of VMMC was centred on strategic planning and coordination of the programme from a district level. Clinical aspects in the delivery of VMMC services typically entailed the delivery of comprehensive HIV-prevention services in addition to pre- and post-operative surgical care.^26,27^ Planning and coordination in terms of VMMC service delivery is an essential role of the policymakers in ensuring correct quality healthcare delivery and provision of VMMC services in line with the national and global quality health standards. This is a vital element to ensure the realisation of the potential health impact of VMMC.^28^

The participants reported experiences of poor preparation and little formal training for the role they were playing in the implementation of VMMC. The poor preparation of healthcare workers, particularly nurses, for clinical roles, is a global phenomenon that has a negative effect on job satisfaction and impedes productivity in the work place.^29,30^ Poor training in the context of VMMC service delivery has many potential negative effects, particularly on the quality of healthcare delivered to male clients, which may influence satisfaction with the health system and ultimately hamper the uptake of VMMC by males.

The experience of service delivery issues was also reported and was attributed to poor infrastructure and staffing shortages. The shortage of staff is also a global problem in general health care.^31,32,33,34^ In terms of VMMC service delivery, similar challenges were cited in Kenya;^35^ however, these were addressed through specific changes in policy and innovative strategies such as task shifting and task sharing by training lower categories of staff to conduct medical circumcisions.^36,37^ In certain settings, in order to meet the high demands of medical circumcisions, the use of assistive devices such as the PrePex and Shang Ring has been proven to be efficient^38,39^; however, the safety and cost-effectiveness of such devices are yet to be established empirically in SA prior to their approval for use.

The policymakers reported that there was a lack of commitment to the VMMC programme, particularly on the part of health personnel working within the Department of Health. This presents a potential problem in terms of the successful implementation of VMMC, particularly because previous studies have shown that the most successful VMMC programmes are those that involve country ownership.^40,41^

Poor role clarification and unclear communication lines were reported among all stakeholders implementing VMMC; the gaps in existing implementation policies such as the unclear roles of all implementers were reported to possibly aggravate this problem. These findings highlight the need for the health system in this context to draw on Kenya’s experience in implementing VMMC by having comprehensive, timed operational plans in order to achieve maximum coverage and yield the full health advantages of VMMC.^26^

The participants’ perceptions of the service centred on the nature of VMMC as a health intervention and the perceived drivers and facilitators for the adoption of VMMC by males. In terms of participants’ perceptions of the nature of VMMC, responses were related to the benefits of VMMC in terms of improving health outcomes by reducing the chances of transmission of HIV and the indirect health benefits to women. These findings concur with those of previous studies^42,43^ in that the health benefits of medical circumcision are a possible motivator for men to undergo VMMC, and represent an important point of entry in demand creation to promote not just sexual health, but overall general health.

Some of the participants also reported that they perceived that the procedure could result in poor understanding regarding the nature of partial protection from HIV leading to possible risky behaviour. Data on men’s understanding of partial protection offered by VMMC vary based on context; in Kenya, it was found that men had an accurate understanding of partial protection;^44^ on the other hand, a study conducted in Swaziland by Grund and Hennink^45^ confirmed the existence of risk compensation following VMMC, thus demonstrating the existence of poor understanding regarding partial protection. The above findings highlight the validity of health workers’ concerns regarding post-VMMC sexual behaviours and the misunderstanding in men regarding partial protection. On the other hand, healthcare workers’ concerns about men’s understanding regarding partial protection could affect the extent to which healthcare workers advocate for men to undergo VMMC. These concerns, therefore, need to be addressed; informing health workers about combination HIV-prevention methods and fostering implementation of these methods could be the first step in dealing with this concern.

Some participants reported having mixed perceptions about the nature of the procedure, especially with regard to safety of male clients in relation to perceived inherent complications related to VMMC surgery. This finding is significant, as it highlights the fact that healthcare workers lack confidence in the health services they provide. This may be attributed to poor training and preparation for the implementation of VMMC, a finding that is also reported in this study. This may be a potential barrier to the full adoption of VMMC as a health strategy by healthcare workers in a clinical setting, and thus needs to be addressed, firstly, by identifying the gaps in their skills and then by providing the necessary training interventions.

Participants, mostly policymakers, reported that their perceived barriers to the uptake of VMMC included the poor mobilisation of medical circumcision by those workers working in clinical settings.

A significant number of participants also reported that males had negative perceptions about the nature of VMMC because of stereotypical norms shaped by culture and society. These perceptions are indicative of the individual male client’s barriers to the acceptability of medical circumcision and serve to highlight the need for engagement with community members and key cultural and religious leaders regarding the medical approach towards male circumcision.

The inadequacy of the health system to meet the high demand was also reported to possibly hinder uptake in certain instances. The lack of financial resources and infrastructure has previously been reported in other settings and has been found to affect the scale-up of VMMC to a certain degree.^46^ Continued donor funding to support governments in regions of priority is thus necessary, and empirical research on the implementation of VMMC in resource-constrained settings is the foundation for attracting such funding.

Persistent demand creation and integration of certain cultural practices into medical circumcision were the perceived drivers for the scale-up of medical circumcision. Currently, mass media and uniform messaging have been used to generate awareness regarding VMMC; prominent community figures such as celebrities and other local influencers have been used. Because, in certain settings, the choice to be circumcised involves deep-seated values, beliefs and motivational factors that vary with ethnic, religious and cultural identities, propagation of VMMC must address these factors in order to effectively generate a demand for medical circumcision.

The study was limited because of the fact that data were only collected at rural clinics and health districts; the findings may therefore not necessarily be applicable to an urban setting. The number of policymakers forming part of the study population was fewer than that of the healthcare workers, leading to a limited population size in this regard. The findings, however, provide important data for policy on the implementation of medical circumcision by healthcare workers in a low-income, rural setting.

## Recommendations

Based on the results from the study, the following recommendations are made:

- The KZN Department of Health should redesign existing policies on the implementation of VMMC to make more explicit the roles and responsibilities of all implementing parties.
- The Department of Health should design VMMC-specific data management and reporting systems, and a clear standard operating procedure should be developed and communicated in this regard.
- The perceived skills’ deficit of all VMMC implementers needs to be identified and addressed through the provision of regular in-service training on all aspects of VMMC, as this could be a key strategy to addressing one of the health system’s potential barriers to the successful scaling-up of VMMC.
- The Department of Health should make specific changes in policy and practice to deal with infrastructure and staffing challenges, such as adopting task-shifting approaches and sourcing additional funding to deal with inadequate infrastructure.
- The Department of Health should also redesign the existing communication strategy for demand creation to include the religious, cultural and traditional dimensions that influence the uptake of VMMC.
- Design a post-VMMC behaviour change intervention to promote positive health-seeking behaviours among men in order to promote the long-term benefits of VMMC – research in the area is recommended.

## Conclusion

The findings of this study suggest that the perceptions of healthcare workers towards VMMC could be potential barriers to healthcare workers advocating medical circumcision. The inadequacy in training and preparation of healthcare workers and policy makers for implementing VMMC could affect uptake of VMMC in that they may not buy in to the service. This may in turn compromise the benefits of successful scale up of VMMC in terms of HIV prevention and the public health benefits thereof.
